# Interaction-Driven Dynamic Fusion for Multimodal Depression Detection: A Controlled Analysis of Gating and Cross-Attention Under Class Imbalance

**DOI:** 10.3390/brainsci16040366

**Published:** 2026-03-28

**Authors:** Kazuyuki Matsumoto, Keita Kiuchi, Hidehiro Umehara, Masahito Nakataki, Shusuke Numata

**Affiliations:** 1 Graduate School of Sciences and Technology for Innovation, Tokushima University, Tokushima 770-8506, Japan; 2 Japan National Institute of Occupational Safety and Health, Japan Organization of Occupational Health and Safety, Kawasaki 214-8585, Japan; kiuchi@h.jniosh.johas.go.jp; 3 Graduate School of Biomedical Sciences, Department of Psychiatry, Tokushima University, Tokushima 770-8506, Japan; umehara.hidehiro@tokushima-u.ac.jp (H.U.); nktk@tokushima-u.ac.jp (M.N.);

**Keywords:** multimodal depression detection, affective pretraining, gated fusion, modality interaction, SigLIP, head pose analysis, PHQ-9, cross-modal weighting

## Abstract

**Highlights:**

**What are the main findings?**

- Cross-attention fusion at the audio integration stage achieved the highest performance (AUC = 0.774; PR-AUC = 0.606) and showed significant superiority over gated and concatenation strategies under class imbalance.
- Visual modality dominance was condition-dependent: SigLIP pretraining yielded positive transfer (+0.018 AUC, p<0.001), whereas audio pretraining produced negative transfer (−0.014 AUC, p=0.004), and intra-visual gating exhibited both balanced weighting and dominance reversal across configurations.

**What are the implications of the main findings?**

- Multimodal depression detection systems should be designed as interaction-driven architectures rather than relying on static fusion taxonomies or assumed semantic superiority.
- Attention-based fusion provides clinically meaningful gains in minority-class detection (PR-AUC improvement), supporting its adoption in imbalanced mental health screening settings.

**Abstract:**

**Background/Objectives:** Multimodal depression detection research has traditionally relied on early or hybrid fusion strategies without systematically analyzing how dynamic fusion mechanisms interact with modality-specific pretraining. Although gated and attention-based architectures are increasingly adopted, their behavior is rarely examined within a structured fusion taxonomy framework. **Methods:** In this study, we conduct a controlled taxonomy-level evaluation of multimodal fusion strategies in a Japanese PHQ-9-annotated depression dataset. We compare four fusion paradigms (concatenation, summation, gated fusion, and cross-attention) across three integration stages, crossed with modality-specific affective pretraining configurations for visual (CMU-MOSI/MOSEI), acoustic (JTES), and textual (WRIME) encoders, yielding 512 experimental conditions. **Results:** The results reveal strong position-dependent effects of fusion strategy. Cross-attention fusion at the audio integration stage achieved the highest mean AUC (0.774) and PR-AUC (0.606), with statistically significant superiority over gated and concatenation-based fusion (Kruskal–Wallis H=86.28, p<0.001). In contrast, fusion effects at the text stage were non-significant in AUC but significant in PR-AUC, highlighting metric-sensitive behavior under class imbalance. Pretraining effects were modality-specific: SigLIP initialization produced significant positive transfer (Δ=+0.018, p<0.001), whereas audio pretraining on JTES resulted in negative transfer (Δ=−0.014, p=0.004), suggesting domain mismatch effects. Gate analysis further revealed condition-dependent modality dominance, including cases of semantic–geometric reversal under joint auxiliary augmentation. **Conclusions:** Our findings suggest that multimodal depression detection systems should not be interpreted through static fusion categories alone. Instead, modality contribution appears to be associated with structured interaction effects between fusion strategy, integration position, and affective pretraining. This work provides a controlled empirical bridge between fusion taxonomy and dynamic modality weighting in clinical multimodal modeling.

## 1. Introduction

Multimodal approaches have become increasingly prominent in depression detection research, leveraging visual, acoustic, and linguistic signals to capture complex behavioral markers. Prior work has demonstrated the effectiveness of pretrained visual semantic embeddings and affective pretraining on datasets such as CMU-MOSI and CMU-MOSEI. These studies often assume that semantically rich visual representations inherently provide stronger predictive signals than geometric facial cues such as head pose.

However, the interaction between affective pretraining and auxiliary multimodal features remains insufficiently understood. In particular, it is unclear whether semantic dominance is stable across multimodal configurations, or whether modality importance dynamically shifts depending on contextual feature composition.

Recent advances in multimodal fusion highlight the importance of gating and attention mechanisms in dynamically weighting modality contributions. Yet, most studies evaluate performance improvements without analyzing how internal modality weighting interacts with model outcomes.

In this study, we investigate the interaction effects between (1) affective pretraining source and (2) auxiliary feature configuration in a multimodal depression classification framework. We integrate SigLIP-based visual semantic embeddings and head pose features within a gated and cross-attention-based temporal pooling network and systematically vary linguistic (E5) and acoustic (OpenSMILE) augmentation under different pretraining regimes spanning four fusion strategies and modality-specific pretraining from CMU-MOSI, CMU-MOSEI (visual), JTES (audio), and WRIME (text), yielding 512 experimental conditions in total.

Rather than focusing solely on overall classification performance, we analyze intra-visual gating behavior and its correspondence with performance changes. Our results reveal condition-dependent modality dominance, including cases of semantic–geometric dominance reversal, demonstrating that multimodal contribution is context-sensitive rather than fixed. These findings challenge the assumption of inherent semantic superiority and suggest that multimodal depression detection systems should be interpreted as interaction-driven integration networks.

**Figure 1:**
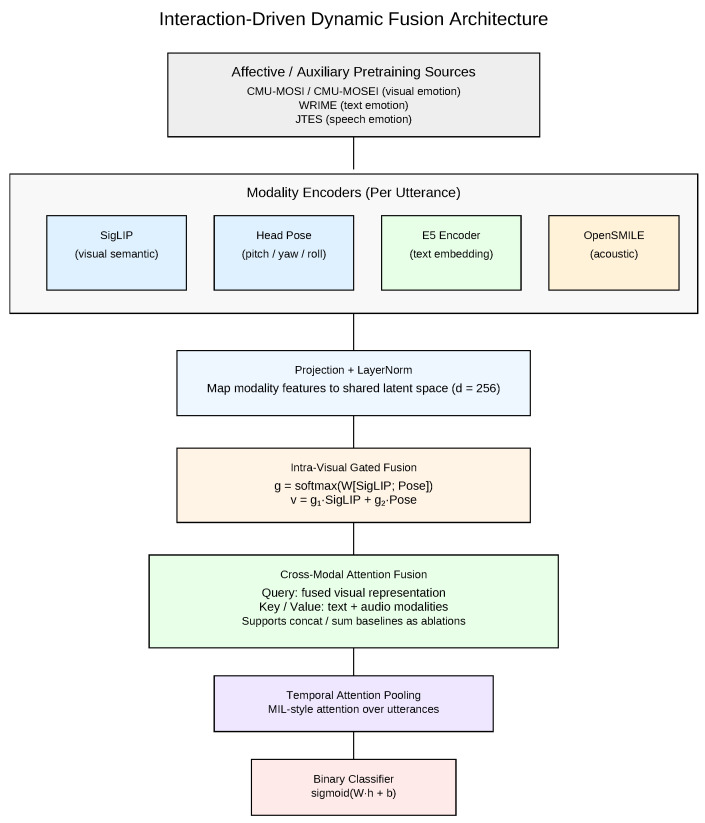
Interaction-driven dynamic fusion architecture. Visual semantic (SigLIP) and geometric (head pose) features are first integrated via intra-visual gated fusion. The resulting visual representation is then combined with optional textual (E5) and acoustic (OpenSMILE) features through cross-modal attention. Temporal attention pooling aggregates utterance-level embeddings into a session-level representation for binary depression classification. Encoders may be initialized using affective or auxiliary pretraining datasets (CMU-MOSI/MOSEI, WRIME, JTES), enabling controlled analysis of transfer-fusion interactions.

To explicitly examine how architectural design interacts with affective pretraining, we introduce an interaction-driven dynamic fusion architecture (Figure 1). Unlike static early or late fusion approaches, the proposed framework separates intra-visual gated integration from cross-modal attention fusion, enabling controlled analysis of modality contribution under different pretraining regimes. This structural decomposition allows us to systematically evaluate how fusion position, fusion mechanism, and transfer initialization jointly influence depression classification performance.

## 2. Related Work

### 2.1. Multimodal Depression Detection

Multimodal depression detection has gained substantial research interest in recent years as a means to improve the objectivity and robustness of automated mental health assessment (Ref. Table 1). Early approaches primarily relied on handcrafted features and conventional classifiers, but recent advances emphasize deep learning models that integrate multiple data modalities such as video, audio, and text to capture complex behavioral and vocal cues associated with depression. For example, Yang et al. proposed multimodal fusion frameworks that integrate facial, voice, and textual cues extracted from clinical interviews to enhance depression diagnosis performance on benchmark datasets such as DAIC-WOZ [1]. These models demonstrate that combining complementary modalities can capture distinct behavioral markers relevant to depression.

Other works have developed specialized neural architectures for multimodal depression classification. Li et al. introduced IMDD-Net, a deep network that fuses visual, acoustic, and textual representations using Kronecker product-based feature integration to improve classification accuracy [2]. Similarly, Nykoniuk et al. explored both early and late fusion strategies for audio and text modalities, showing that multimodal architectures consistently outperform unimodal baselines [3]. Multi-head attention and graph convolution networks have also been employed to handle temporal dependencies and cross-modal interactions, indicating the diversity of modeling choices in this domain [4].

Despite these developments, most existing methods can be categorized as either feature-level fusion or attention-based integration without explicitly disentangling the contribution of pretrained external representations. Additionally, while some studies report high classification performance, systematic comparisons of fusion mechanisms under controlled feature configurations remain limited.

### 2.2. Affective and Multimodal Representation Learning

Representation learning has played a central role in multimodal modeling, particularly in emotion recognition and related behavioral analysis tasks. Deep multimodal representation learning seeks to project heterogeneous modality signals into a shared latent space that captures cross-modal correlations [5]. Such approaches have proven effective in tasks like sentiment analysis, video classification, and event detection, and have informed subsequent multimodal depression detection research.

In the context of affective computing, pretrained models such as large-scale vision–language networks and pretrained text/audio encoders have demonstrated strong generalization to downstream tasks. However, most depression detection studies either use pretrained components ad hoc or train end-to-end without quantifying the specific benefits of pretrained affective representations. In contrast, our study explicitly evaluates affective pretraining transfer by comparing models trained with and without pretrained representations while keeping fusion strategies constant.

### 2.3. Fusion Strategies in Multimodal Systems

Fusion strategies in multimodal systems have traditionally been categorized into early, intermediate, late, and hybrid fusion paradigms [6,7].

Early fusion refers to feature-level concatenation prior to model learning, while late fusion aggregates modality-specific predictions at the decision level. Intermediate fusion integrates modality representations within shared latent spaces during learning, and hybrid approaches combine multiple strategies across different integration stages.

Such taxonomies have been systematically organized in several survey studies across multimodal learning and biomedical applications [6,8], where pixel-level, feature-level, and decision-level fusion correspond to early, intermediate, and late fusion, respectively. These classifications provide a conceptual foundation for understanding how modality interactions are structurally modeled.

In recent years, many multimodal architectures have moved beyond static concatenation toward dynamic fusion mechanisms that adapt modality contributions according to context. Attention-based fusion learns context-dependent weighting through query–key–value interactions, whereas gated fusion introduces sigmoid-based control signals that regulate modality influence. Several contemporary models adopt hybrid dynamic fusion strategies combining both mechanisms, demonstrating superiority over simple concatenation in tasks ranging from financial time-series analysis to fake news detection [9,10].

In affective computing and sentiment analysis, hierarchical gated fusion and dynamic modality control mechanisms have also been widely adopted to dynamically adjust modality importance at the utterance level [11].

Systematic evaluations in biomedical multimodal surveys further suggest that intermediate fusion and attention-based inter-modality weighting are particularly promising due to their ability to learn joint representations while preserving modality-specific characteristics [12]. Attention-based fusion has increasingly been treated as an independent category in sentiment and emotion recognition surveys, where modality contribution varies across utterances and contextual weighting becomes critical [11].

Despite these advances, few studies have conducted controlled, large-scale comparisons of fusion strategies under systematically varied pretraining and modality configurations. Our study aims to address this gap by systematically evaluating 512 controlled conditions across fusion positions and pretraining regimes, enabling an analysis of interaction effects between architectural design and affective pretraining. In contrast to prior works that have primarily reported aggregate performance improvements, we explicitly analyze how dynamic modality weighting interacts with affective pretraining. By linking architectural design with representational transfer effects, our framework provides a structured perspective on interaction-driven multimodal integration.

## 3. Materials and Methods

### 3.1. Dataset and Task Definition

The primary dataset used in this study is a Japanese multimodal interview dataset annotated with depression tendency labels based on the Patient Health Questionnaire-9 (PHQ-9). The dataset was introduced in our previous work [13], where data from multimodal interview sessions were collected and annotated for depressive tendency using clinically grounded scoring criteria.

Depression tendency labels were derived from PHQ-9 scores [14], a widely validated self-report measure of depressive symptom severity, and were binarized into two categories (depressed vs. non-depressed) using a clinically motivated threshold.

The task in this study is formulated as binary classification:

(1) y∈{0,1},

where y=1 denotes the depressed group and y=0 denotes the non-depressed group.

To ensure subject independence, data splits were constructed such that no participant appeared in multiple subsets. Model performance was evaluated using AUC and PR-AUC as primary metrics, with F1-score and balanced accuracy reported as supplementary measures.

### 3.2. External Pretraining Datasets

To investigate the transfer effect of affective pretraining, we utilized two widely used English multimodal emotion datasets:

- **CMU-MOSI** [15]
- **CMU-MOSEI** [16]

In addition, to evaluate the effect of Japanese-domain affective pretraining on the acoustic and linguistic encoders, we employed two Japanese emotional corpora:

- **JTES (Japanese Twitter-based Emotional Speech)** [17]: A Japanese speech corpus annotated with emotional categories. The acoustic encoder (OpenSMILE feature projection layers) was pretrained on emotion classification using JTES before fine-tuning on the target depression dataset.
- **WRIME (Word-level Relevance and Intensity of Meaning in Emotion)** [18]: A Japanese social media sentiment/emotion annotation corpus. The text encoder (E5 projection layers) was pretrained on sentiment prediction using WRIME before fine-tuning.

These corpora share the Japanese language domain with the target dataset, allowing us to assess whether domain-matched emotional pretraining provides a transfer advantage over English-language corpora or random initialization.

### 3.3. Multimodal Feature Extraction

#### 3.3.1. Visual Features

The visual modality consists of two complementary components:

(1) SigLIP-Based Semantic Visual Embeddings

Frame-level embeddings were extracted using a pretrained SigLIP vision–language model. Given an input frame $x_{t}$, the visual embedding is defined as

(2) $$v_{t}^{\mathrm{siglip}}=f_{\mathrm{SigLIP}}\left(x_{t}\right).$$

Temporal aggregation was performed via mean pooling:

(3) $$v^{\mathrm{siglip}}=\frac{1}{T}\sum_{t=1}^{T}v_{t}^{\mathrm{siglip}}.$$

(2) Head Pose Features

Head pose features (pitch, yaw, roll) were extracted for each frame:

(4) $$v_{t}^{\mathrm{pose}}=\left[{\mathrm{pitch}}_{t},{\mathrm{yaw}}_{t},{\mathrm{roll}}_{t}\right].$$

Similarly, temporal pooling was applied:

(5) $$v^{\mathrm{pose}}=\frac{1}{T}\sum_{t=1}^{T}v_{t}^{\mathrm{pose}}.$$

#### 3.3.2. Linguistic Features

Textual utterances were encoded using pretrained E5 embeddings:

(6) $$v^{\mathrm{text}}=f_{E5}\left(u\right),$$

where *u* denotes the transcribed utterance.

#### 3.3.3. Acoustic Features

Acoustic features were extracted using OpenSMILE descriptors:

(7) $$v^{\mathrm{audio}}=f_{\mathrm{OpenSMILE}}\left(a\right),$$

where *a* represents the audio signal segment.

### 3.4. Interaction-Driven Dynamic Fusion Architecture

Figure 1 illustrates the proposed architecture. Each modality encoder (SigLIP, head pose, E5, OpenSMILE) is first projected into a shared latent space. Visual semantic and geometric cues are integrated via intra-visual gated fusion, after which cross-modal attention dynamically combines visual, acoustic, and textual representations. Temporal attention pooling then aggregates utterance-level embeddings into a session-level representation for classification.

Before projection, visual embeddings are normalized using layer normalization [19] to reduce scale discrepancies between semantic and geometric representations.

#### 3.4.1. Feature Projection

Each modality embedding was projected into a shared latent space:

(8) $${\tilde{v}}^{m}=W_{m}v^{m}+b_{m},$$

where m∈{siglip,pose,text,audio}, and $v^{m}$ denotes the raw feature vector for modality *m* as defined in Equations (2)–(7).

Implementation Details.

The large-scale feature extractors (SigLIP vision–language model, multilingual E5, OpenSMILE) were used exclusively for feature extraction; their backbone weights were not updated during any training phase. Only the modality-specific projection MLPs were subject to pretraining and subsequent fine-tuning.

Specifically, the SigLIP projection consists of a single Linear($d_{s}\to 256$) + ReLU + Dropout layer; the audio projection is a 2-layer MLP [Linear(88→128) → BatchNorm → ReLU → Dropout] × 2; and the text projection is a 2-layer MLP [Linear(1024→256) → LayerNorm → GELU → Dropout] × 2. All projection layers were fine-tuned end-to-end during downstream depression classification training regardless of initialization.

The shared hidden dimension was set to d=256, chosen as a practical balance between representational capacity and the risk of overfitting given the limited dataset size (*n* = 74 subjects). When the pretrained condition was active, the corresponding projection layer was initialized from the affective pretraining checkpoint (CMU-MOSI/MOSEI for SigLIP, JTES for audio, WRIME for text) prior to downstream fine-tuning.

#### 3.4.2. Fusion Strategies

Table 2 summarizes the four fusion strategies evaluated in this study, including their operation, approximate parameter overhead per fusion stage, and interpretability mechanism.

We evaluated four fusion mechanisms:

(1) Concatenation Fusion
  (9) $$z=[{\tilde{v}}^{\mathrm{siglip}};{\tilde{v}}^{\mathrm{pose}};{\tilde{v}}^{\mathrm{text}};{\tilde{v}}^{\mathrm{audio}}].$$
(2) Summation Fusion
  (10) $$z=\sum_{m}{\tilde{v}}^{m}.$$
(3) Gated Visual Fusion

Within the visual branch, SigLIP and head pose features were combined using a gating mechanism:

(11) $$g=\sigma (W_{g}\left[{\tilde{v}}^{\mathrm{siglip}};{\tilde{v}}^{\mathrm{pose}}\right]+b_{g}),$$

This gating formulation is conceptually related to gated multimodal fusion approaches that dynamically regulate modality contribution [20].

(12) $${\tilde{v}}^{\mathrm{visual}}=g⊙{\tilde{v}}^{\mathrm{siglip}}+\left(1-g\right)⊙{\tilde{v}}^{\mathrm{pose}},$$

where σ denotes the sigmoid function and ⊙ denotes element-wise multiplication.

The fused visual embedding was then combined with linguistic and acoustic features.

(4) Cross-Attention Fusion

A cross-attention mechanism is applied at each fusion stage, where the primary modality representation serves as the query and the complementary modality provides key–value pairs:

Given two modality representations ${\tilde{v}}^{a}$ (primary) and ${\tilde{v}}^{b}$ (complementary), cross-attention fusion computes

(13) $$Q=W_{Q}{\tilde{v}}^{a},\;K=W_{K}{\tilde{v}}^{b},\;V=W_{V}{\tilde{v}}^{b}$$

(14) $$\alpha =\mathrm{softmax}\;\left(\frac{QK^{⊤}}{\sqrt{d_{k}}}\right)$$

(15) c=αV

(16) $${\tilde{v}}^{\mathrm{fused}}=W_{O}\left[{\tilde{v}}^{a}\;;\;c\right]$$

where $W_{Q},W_{K},W_{V}\in R^{d_{k}\times d}$, and $W_{O}\in R^{d\times 2d}$ are learned projection matrices, $d_{k}$ denotes the key/query dimension, [ ; ] denotes concatenation, and ⊤ denotes matrix transposition. The attention weight $\alpha \in R^{1\times T_{\mathrm{utt}}}$ provides a per-utterance soft weighting of the complementary modality, analogous to the gate *g* in Equation (11), and can be used for post hoc interpretability analysis of inter-modal contribution.

#### 3.4.3. Attention-Based Temporal Aggregation

Utterance-level representations were aggregated using attention-based pooling. Given a sequence of hidden states ${\left\{h_{t}\right\}}_{t=1}^{T}$, attention scores were computed as

(17) $$\alpha_{t}=\frac{\exp(w^{⊤}h_{t})}{\sum_{k=1}^{T}\exp\left(w^{⊤}h_{k}\right)}.$$

The aggregated representation was then obtained as

(18) $$h=\sum_{t=1}^{T}\alpha_{t}h_{t}.$$

This mechanism is conceptually related to attention-based multiple instance learning [21], where instance-level representations are softly weighted to form a global prediction.

#### 3.4.4. Session-Level Classification

The aggregated session-level representation *h* obtained via attention-based temporal pooling (Equation (18)) was fed into a binary classification layer:

(19) $$\hat{y}=\sigma \left(W_{o}h+b_{o}\right),$$

where σ denotes the sigmoid function, and $W_{o}$, $b_{o}$ are learned parameters.

### 3.5. Controlled Pretraining Evaluation

To isolate the transfer effects of affective pretraining across modalities, we compared the following initialization conditions for each modality encoder independently:

- SigLIP visual encoder: pretrained on CMU-MOSEI/CMU-MOSI vs. random initialization.
- Audio encoder (OpenSMILE projection): pretrained on JTES vs. random initialization [22].
- Text encoder (E5 projection): pretrained on WRIME vs. random initialization [23].

By independently varying each modality’s pretraining state, the full evaluation covers $2^{3}=8$ pretraining combinations, crossed with $4^{3}=64$ fusion strategy combinations, yielding 512 experimental conditions in total. All architectural components and training hyperparameters were held constant across conditions.

### 3.6. Training Objective

Models were optimized using weighted binary cross-entropy loss to address class imbalance. Let $w_{\mathrm{pos}}=n_{\mathrm{neg}}/n_{\mathrm{pos}}$ denote the per-fold positive class weight, computed from the training fold labels at each cross-validation fold (overall ratio ≈1:4.7). The loss is calculated by Equation (20)

(20) $$L=-\frac{1}{N}\sum_{i}\left[w_{\mathrm{pos}}\;y_{i}\log{\hat{y}}_{i}+\left(1-y_{i}\right)\log\left(1-{\hat{y}}_{i}\right)\right].$$

This weight directly rescales the loss contribution of minority-class (depressed) samples relative to the majority class. No resampling was applied. Focal loss (with α=0.25, γ=2.0, combined with $w_{\mathrm{pos}}$) was also implemented but was not used in the primary 512-condition evaluation in order to maintain a single consistent optimization setting across all conditions.

Optimization was performed using AdamW ($\mathrm{lr}=10^{-3}$, weight decay $=10^{-4}$) with gradient clipping (max norm =1.0) and early stopping based on validation AUC.

In the preparation of this study, artificial intelligence (AI)-assisted tools were used in the following capacities: Claude (Sonnet 4.6; Anthropic, San Francisco, CA, USA) was used to assist in generating source code for machine learning algorithms and producing figures. ChatGPT (GPT-5.3 Instant; OpenAI, San Francisco, CA, USA) was used to assist with portions of the literature review, text summarization, and proofreading. All AI-generated content was reviewed and edited by the authors, who take full responsibility for the accuracy and integrity of the reported work.

## 4. Results

Given the class imbalance in the dataset (depressed: n=13; non-depressed: n=61; ratio ≈ 1:5), we report both AUC (area under the ROC curve) and PR-AUC (area under the precision–recall curve) as primary evaluation metrics. PR-AUC is particularly informative under class imbalance, as it directly reflects the model’s ability to identify the minority (depressed) class.

### 4.1. Overall Performance Across Fusion Strategies

Table 3 reports AUC values for the concat fusion strategy under varying SigLIP pretraining conditions, consistent with the prior findings. As summarized in Table 4 and Table 5, fusion strategy effects exhibit clear position-dependent characteristics. At the vis_fusion stage, concatenation achieved the highest mean AUC (0.769), with a moderate effect size ($\eta^{2}=0.203$), whereas at the audio_fusion stage, cross-attention significantly outperformed all other strategies (mean AUC = 0.774, $\eta^{2}=0.679$).

Notably, while AUC differences at the text_fusion stage were non-significant (H=0.89, p=0.828), the PR-AUC analysis revealed a significant effect (H=16.08, p=0.001, $\eta^{2}=0.127$), indicating that fusion strategy selection influences minority-class detection even when overall ROC-based discrimination appears stable.

Key observation: Cross-attention fusion achieved the highest mean AUC at the audio integration stage (0.7736), significantly outperforming gated fusion (Δ=+0.041, p<0.001) and concat fusion (Δ=+0.010, p=0.025). Notably, the effect of fusion strategy was not significant at the text_fusion position (H=0.89, p=0.83), suggesting that the choice of fusion method matters primarily for acoustic integration, whereas text integration is comparatively robust to fusion design.

Regarding vis_fusion, concat remained the best-performing strategy (0.7694), consistent with the original findings. The Kruskal–Wallis test was significant (*H* = 25.73, *p* < 0.001) but the effect size was smaller than for audio_fusion.

Furthermore, a notable dissociation is observed in the interaction between audio_fusion and the number of pretrained modalities: whereas gated and sum fusion performance degrades monotonically as more modalities are pretrained, cross-attention fusion improves with additional pretraining. This interaction suggests that cross-attention fusion is better equipped to leverage pretrained representations, possibly because the query-key-value mechanism can selectively integrate high-quality pretrained features.

To assess the effect of fusion strategy on classification performance, we applied the Kruskal–Wallis test across the four strategies at each fusion position. The effect was significant at the vis_fusion stage (H=25.73, p<0.001) and strongly significant at the audio_fusion stage (H=86.28, p<0.001), but non-significant at the text_fusion stage (H=0.89, p=0.828).

### 4.2. Effect of Affective Pretraining

We extended the pretraining analysis to cover all three modality encoders. Table 6 summarizes the main effects of each modality’s pretraining status on AUC, estimated via Mann–Whitney U tests across 256 matched pairs.

SigLIP pretraining remained the only modality-specific pretraining condition to yield a statistically significant positive effect (Δ=+0.018, p<0.001), consistent with the original findings regarding MOSEI pretraining benefits.

Unexpectedly, audio pretraining on JTES showed a significant negative transfer effect (Δ=−0.014, p=0.004). JTES is a Japanese emotional speech corpus collected from Twitter-based recordings, which may differ substantially from the naturalistic, clinically elicited speech in the depression interview dataset. Domain mismatch between the pretraining source and the target distribution may contribute to this degradation.

Text pretraining on WRIME yielded no significant effect (Δ=−0.005, p=0.229), suggesting that sentiment-based pretraining of the E5 projection layer does not meaningfully improve or degrade depression classification. Notably, when all three modalities are pretrained simultaneously, the net effect cancels out (Δ≈0), indicating that the positive SigLIP effect and negative Audio effect offset each other.

Interestingly, MOSEI pretraining demonstrated more stable performance (lower variance) compared to random initialization in several fusion settings, particularly under summation fusion.

### 4.3. Additional Feature Conditions

We further analyzed the effect of additional features (OpenSMILE, E5, or none). Across all fusion strategies, the inclusion of linguistic (E5) and acoustic (OpenSMILE) features produced modest improvements in F1-score in some configurations but did not consistently improve AUC.

Notably, the gated fusion architecture applies gating only within the visual branch (SigLIP and head pose), while additional features are concatenated after visual fusion. This architectural design may limit the adaptive weighting effect for non-visual modalities.

### 4.4. Summary of Key Findings

The main empirical findings are summarized as follows:

1. Concatenation-based fusion achieved competitive or superior performance compared to gated fusion.
2. SigLIP pretraining yielded a significant positive transfer effect (Δ=+0.018, p<0.001), whereas audio pretraining on JTES produced significant negative transfer (Δ=−0.014, p=0.004), and text pretraining showed no significant effect (p=0.229).
3. Additional linguistic and acoustic features provided limited incremental gains.
4. Cross-attention fusion at the audio_fusion stage achieved the highest mean AUC (0.774) and PR-AUC (0.606), significantly outperforming all other strategies (*H* = 86.28, *p* < 0.001).
5. Fusion strategy selection exhibited position-dependent effects: significant at the vis_fusion and audio_fusion stages, but non-significant in AUC at the text_fusion stage (p=0.828).
6. A divergence between AUC and PR-AUC at the text_fusion stage revealed that cross-attention fusion provides clinically meaningful minority-class detection gains not captured by AUC alone $\left(\Delta_{PR-AUC}=+0.028,p<0.001\right)$, underscoring the importance of evaluating both metrics under class imbalance.

Overall, these results are consistent with the view that fusion architecture and pretraining strategy interact as interdependent design choices: cross-attention fusion showed the strongest synergy with pretrained representations under the present conditions, while modality selection for pretraining requires careful domain alignment.

### 4.5. Gate Weight Analysis

To investigate modality dominance within the visual branch, we analyzed the difference between SigLIP and head pose gate weights at the subject level:

(21) $$\Delta =g_{\mathrm{SigLIP}}-g_{\mathrm{Pose}}.$$

A positive Δ indicates the dominance of SigLIP representations, whereas a negative value indicates greater weighting of head pose features. Across experimental conditions, gating behavior exhibited substantial variability.

Strong SigLIP Dominance

In the absence of additional features under MOSEI pretraining, the gate exhibited near-deterministic selection of SigLIP representations (Δ=0.9378). A paired-sample *t*-test confirmed a highly significant difference between SigLIP and head pose weights (t=73.36, $p<10^{-69})$.

Similarly, under the no-pretraining condition, strong SigLIP dominance was observed (Δ=0.8056, t=18.31, $p<10^{-28})$.

When linguistic features (E5) were introduced, SigLIP remained strongly dominant across both pretraining datasets. Under MOSEI pretraining, Δ=0.8080 (t=34.26, *p* < $10^{-46}$), and under MOSI pretraining, Δ=0.7516 (t=18.99, $p<10^{-29})$.

Balanced Integration

In contrast, under the *OpenSMILE + MOSEI* condition, no significant dominance was observed (Δ=0.0315, t=0.30, p=0.76). This indicates that semantic and geometric visual cues were weighted approximately equally in this configuration.

Reversal of Dominance

Notably, under the combined *E5_OpenSMILE + MOSI* condition, gating behavior reversed, with head pose receiving greater weight than SigLIP (Δ=−0.3754, t=−6.72, $p<10^{-8})$. This inversion suggests that the multimodal context and pretraining source jointly influence intra-visual weighting dynamics.

Moderate SigLIP Preference

Under the *OpenSMILE + MOSI* condition, SigLIP retained moderate dominance (Δ=0.4307, t=4.69, $p<10^{-5})$. Similarly, in the *none + MOSI* configuration, a smaller but statistically significant SigLIP preference was observed (Δ=0.2702, t=2.63, p=0.010).

Summary of Findings

Overall, the gating mechanism does not universally collapse to semantic dominance. Instead, modality weighting is highly condition-dependent and influenced by both external affective pretraining and auxiliary feature composition. While SigLIP representations frequently receive dominant weights, balanced integration and even reversal of dominance occur under specific multimodal configurations. These results indicate that gating behavior reflects dynamic interaction effects rather than a fixed preference for pretrained semantic representations.

**Figure 2:**
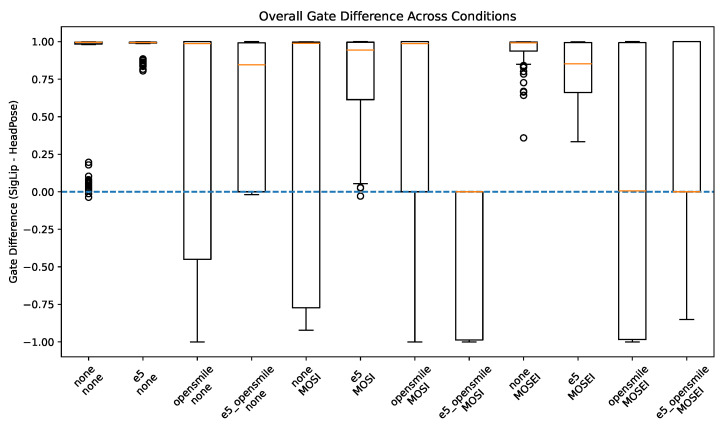
Overall distribution of gate weight differences $\left(\Delta =w_{\mathrm{SigLIP}}-w_{\mathrm{pose}}\right)$ across all combinations of pretraining and auxiliary features. Positive values indicate semantic dominance, whereas negative values indicate geometric dominance. The dashed horizontal line denotes equal weighting. Substantial variability and dominance reversal under specific configurations demonstrate interaction effects between affective pretraining and multimodal feature composition. The statistical significance per condition is reported in Section 4.5.

As shown in Figure 2, gate weighting exhibits substantial variability across pretraining and auxiliary feature configurations, including cases of balanced integration and dominance reversal.

**Figure 3:**
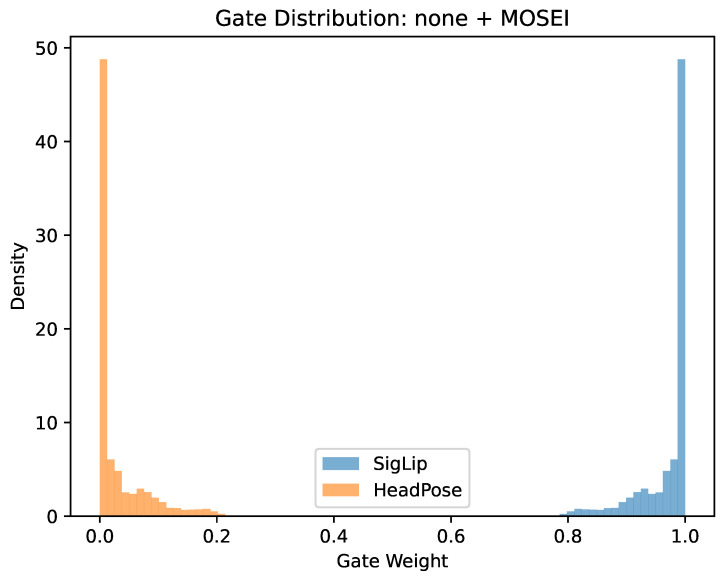
Distribution of gate weights for SigLIP and head pose features under the none + MOSEI configuration. Semantic embeddings receive near-deterministic weighting, while head pose contributions are suppressed. This condition illustrates extreme semantic dominance.

Figure 3 illustrates a representative condition (none + MOSEI), where near-deterministic semantic dominance is observed. This extreme case contrasts with the dominance reversal observed in E5 + OpenSMILE + MOSI, emphasizing interaction-driven variability.

Hierarchical dynamic gate distributions and confusion matrix comparisons under the best-performing cross-attention configuration are provided in the Supplementary Materials (Figures S1 and S2).

Additional qualitative analysis of embedding geometry is provided in Appendix A.

In summary, the gate weight analysis revealed that semantic visual representations (SigLIP) dominated geometric head pose features in gated weighting under most configurations, although dominance was condition-dependent, exhibiting balanced integration and reversal under specific multimodal contexts.

## 5. Discussion

### 5.1. Condition-Dependent Visual Modality Dominance

The gate analysis revealed that intra-visual modality weighting was highly condition-dependent rather than universally dominated by semantic representations. In several configurations (e.g., none + MOSEI, none + none, E5 + MOSEI), the gating mechanism exhibited strong SigLIP dominance, which is consistent with the interpretation that pretrained semantic visual embeddings provide highly discriminative signals for depression classification under these conditions. These results align with the hypothesis that semantic abstraction learned from large-scale data captures clinically relevant behavioral patterns beyond low-level motor cues, although this remains an interpretive account rather than a demonstrated mechanism.

However, this dominance was not universal. Under the OpenSMILE + MOSEI condition, the gate exhibited balanced weighting between SigLIP and head pose features, and under the E5_OpenSMILE + MOSI condition, dominance was reversed in favor of head pose representations.

These findings indicate that the gating mechanism does not simply converge to pretrained semantic features, but instead assigns weights that vary substantially with the broader multimodal configuration. Whether this reflects true redistribution of predictive burden or is an artifact of optimization dynamics remains an open question requiring further investigation.

### 5.2. Interaction Between Pretraining and Auxiliary Features

The observed reversals are consistent with an interaction effect between the affective pretraining source and auxiliary feature composition. Specifically, when linguistic and acoustic features are jointly introduced under MOSI pretraining, semantic dominance weakens and geometric cues become relatively more influential. This pattern is consistent with the interpretation that auxiliary modalities may alter the marginal predictive contribution of visual semantics, potentially associated with a redistribution of learned weighting across the multimodal system. However, this remains a descriptive observation; the precise mechanism underlying the shift cannot be determined from the current analysis.

From a modeling perspective, this behavior is consistent with gating functioning as a context-sensitive weighting mechanism rather than a fixed selector. From a clinical perspective, it raises the possibility that depression-related visual cues may shift between semantic and motor characteristics depending on the presence of complementary linguistic or prosodic information, though causal claims in this direction would require controlled experimental designs.

### 5.3. Implications for Multimodal Depression Modeling

These findings carry three important implications, to be interpreted within the constraints of the current experimental setting:

1. Representation quality alone does not appear to determine dominance under the observed conditions; multimodal context is associated with shifts in intra-branch weighting.
2. Affective pretraining can be associated with stabilized semantic dominance but does not universally override geometric cues across all configurations.
3. Gating behavior in clinical multimodal systems may reflect higher-order interaction effects rather than simple feature importance, though causal interpretation of this behavior requires further investigation.

Overall, our results suggest that multimodal depression detection involves context-dependent interdependencies across modalities within this experimental framework, and that visual semantic and motor representations may contribute differentially depending on the configuration.

### 5.4. Gate–Performance Interaction Dynamics

The gate analysis revealed substantial condition-dependent variability in intra-visual modality dominance. These dominance patterns were associated with shifts in classification performance; the following observations are descriptive and do not establish causal relationships.

Under MOSEI pretraining without auxiliary features, the gating mechanism exhibited near-deterministic semantic dominance $\left(\Delta =0.9378,\;p<10^{-69}\right)$, coinciding with strong classification performance. When linguistic features were introduced (E5 + MOSEI), semantic dominance remained high (Δ=0.8080), and performance remained competitive. In contrast, under the *OpenSMILE + MOSEI* configuration, visual dominance disappeared (Δ=0.0315, p=0.76), indicating balanced weighting between semantic and geometric cues; this pattern is consistent with the interpretation that acoustic augmentation is associated with altered weighting within the visual branch, although the mechanism is not established by the current analysis. Under the *E5_OpenSMILE + MOSI* condition, dominance reversed in favor of head pose features $\left(\Delta =-0.3754,\;p<10^{-8}\right)$, consistent with the view that multimodal context and pretraining source jointly influence intra-visual weighting rather than enforcing fixed semantic superiority.

These findings indicate that gating behavior is associated with multimodal performance characteristics in a condition-dependent manner; visual semantic dominance is not universally observed, and modality weighting varies with broader feature composition and pretraining context.

Taken together, the gate–performance correspondence reveals a descriptive pattern: conditions with high SigLIP gate dominance (none+MOSEI, E5 + MOSEI) coincide with competitive AUC values (Table 3), while balanced or reversed weighting conditions (openSMILE + MOSEI, E5 + OpenSMILE + MOSI) correspond to configurations where neither visual modality alone provides a stable predictive signal. This alignment between learned modality weighting and classification outcomes is consistent with the view that gate behavior reflects condition-specific weighting patterns, though whether this constitutes a mechanistic account requires further investigation.

### 5.5. Divergence Between AUC and PR-AUC at the Text Fusion Stage

A notable divergence between AUC and PR-AUC was observed at the text_fusion stage. The divergence observed between Table 4 and Table 5 underscores the importance of evaluating fusion strategies under both ROC-based and precision–recall-based metrics, particularly in imbalanced clinical datasets.

Although the Kruskal–Wallis test revealed no significant difference across fusion strategies in terms of AUC (*H* = 0.89, *p* = 0.828), a significant effect emerged when PR-AUC was used as the evaluation criterion (*H* = 16.08, *p* = 0.001). Cross-attention fusion achieved the highest PR-AUC among all text_fusion strategies (mean =0.579), significantly outperforming concatenation fusion (Δ=+0.028, Mann–Whitney *U* test, p<0.001).

This divergence can be attributed to the nature of the two metrics under class imbalance. In the present dataset, the depressed group accounts for only 17.6% of participants (13 out of 74), making the random-baseline PR-AUC approximately 0.176. AUC, which, being derived from the ROC curve, is relatively robust to class imbalance but may mask differences in minority-class detection performance. PR-AUC, by contrast, directly captures the model’s ability to correctly identify depressed individuals while controlling for false positives, and is therefore more sensitive to differences in minority-class discrimination.

These results suggest that cross-attention fusion at the text integration stage provides a clinically meaningful advantage in depression detection that is not captured by AUC alone. Precision–recall curves aggregated across all folds for the cross-attention model and concatenation baseline are provided in the Supplementary Materials (Figure S3). From a clinical deployment perspective, where minimizing missed detections (false negatives) is paramount, PR-AUC may be a more appropriate primary metric than AUC. Furthermore, the consistent superiority of cross-attention fusion across both audio_fusion ($\Delta_{\mathrm{AUC}}=+0.041$, $\Delta_{PR-AUC}=+0.085$ vs. gated) and text_fusion ($\Delta_{PR-AUC}=+0.028$ vs. concat) reinforces the recommendation to adopt attention-based integration as the default fusion strategy in multimodal depression detection systems.

The observed interaction in which cross-attention performance improves with additional pretraining, while gated and sum fusion degrade, is consistent with the general principle that attention-based modules—having a larger parameter count and greater expressive capacity—tend to benefit more from high-quality pretrained initialization, particularly when in-domain training data are limited. This interpretation is descriptive rather than causal: the observed correlation between pretraining benefit and fusion strategy does not establish that parameter count is the sole mechanism, and capacity-matched comparisons would be required to confirm this hypothesis.

## 6. Conclusions

This study investigated how affective pretraining and auxiliary multimodal features influence intra-visual modality weighting and classification performance in multimodal depression detection, within a controlled experimental framework using a Japanese PHQ-9-annotated interview corpus (n=74; depressed: n=13, non-depressed: n=61).

Through systematic evaluation across pretraining sources and auxiliary feature configurations, we observed that visual semantic dominance is not universally stable within the scope of this study. Instead, gating dynamics varied substantially across conditions, including cases of dominance reversal under specific multimodal contexts. These gating shifts were aligned with changes in classification performance, indicating that modality contribution is associated with interaction effects rather than fixed representational strength.

Importantly, our findings challenge the assumption that pretrained semantic embeddings inherently dominate geometric facial cues. While semantic representations frequently exhibited strong influence, their contribution depended on broader multimodal composition and pretraining source. Acoustic and linguistic augmentation could rebalance or invert intra-visual weighting, suggesting that multimodal systems operate as context-sensitive integration networks under these experimental conditions.

These results emphasize the need to analyze modality interaction mechanisms—not only performance metrics—when designing clinical multimodal models. Future work should further investigate dynamic gating strategies and domain-adaptive pretraining to better understand how representational context shapes predictive behavior.

Overall, this study provides preliminary empirical evidence that multimodal depression detection performance is associated with structured interaction effects across modalities and pretraining regimes, rather than isolated modality superiority. Replication on larger and more diverse datasets is necessary to assess the generalizability of these findings.

Regarding practical selection criteria, cross-attention fusion is recommended as the default strategy when pretrained representations are available, given its consistent PR-AUC advantage in minority-class detection. Concatenation fusion offers a computationally lighter alternative (approximately half the additional parameter count of cross-attention per fusion stage) when pretraining is absent or computational resources are constrained.

## 7. Limitations

Several limitations of this study should be acknowledged.

First, the primary dataset consists of Japanese interview sessions with PHQ-9-based binary labels (n=74; depressed: n=13, non-depressed: n=61). The relatively small sample size limits the statistical power of the reported interaction effects and the generalizability of the findings to other populations, languages, or clinical settings. Although subject-independent cross-validation was employed to mitigate overfitting, the observed AUC and PR-AUC values should be interpreted as preliminary estimates within these specific experimental conditions. Replication on larger and more diverse datasets is necessary before the reported interaction effects can be considered robust.

Second, PHQ-9 scores were binarized into a two-class depression label using a clinically motivated threshold. While PHQ-9 is a widely validated instrument, this binarization reduces the richness of the original severity spectrum and may limit clinical applicability, where graded severity assessment is often more informative than binary screening. Future work should investigate ordinal or regression-based modeling approaches that preserve continuous severity information.

Third, the comparison among concatenation, summation, gated fusion, and cross-attention involves modules that differ in expressive capacity and parameter count. Summation adds no parameters; concatenation adds approximately $2d^{2}$ parameters per stage; gated fusion adds ∼2$d^{2}+d$; and cross-attention adds ∼4$d^{2}$ (i.e., approximately twice the additional parameters of concatenation, with d=256). The observed performance advantage of cross-attention may therefore partly reflect greater model capacity and flexibility, rather than a purely superior fusion principle. Capacity-matched comparisons—for example, matching total parameter counts across fusion types by adjusting hidden dimensions—would be required to fully disentangle fusion-principle effects from capacity effects. This limitation does not invalidate the practical findings, but constrains the theoretical interpretation.

Fourth, the gate weight and attention weight analyses are descriptive characterizations of learned weighting behavior under the specific experimental conditions of this study. These analyses demonstrate statistical correspondence between modality weighting patterns and classification outcomes, but do not establish mechanistic causality. Causal interpretation would require controlled interventions such as ablation of individual feature components or counterfactual analysis. All claims regarding modality dominance and pretraining interaction effects should be understood as empirical associations observed within this framework.

Fifth, affective pretraining was conducted using English-language datasets (CMU-MOSI and CMU-MOSEI) for the visual encoder, while the target dataset is Japanese. Regarding cross-lingual and cross-cultural domain mismatch: the SigLIP visual encoder exhibited positive transfer despite the English-to-Japanese mismatch, likely because facial appearance and expression features are relatively language-agnostic. In contrast, the audio pretraining corpus (JTES), although Japanese, produced significant negative transfer, suggesting that recording conditions (Twitter-sourced emotional speech vs. controlled clinical interviews) represent a more critical domain boundary than linguistic alignment for acoustic features. The text pretraining corpus (WRIME), while linguistically matched, showed no significant effect, possibly because social media sentiment discourse differs substantially from clinical interview language. These observations suggest that domain alignment in terms of recording conditions and discourse context may be more consequential than linguistic alignment, particularly for acoustic and textual modalities. Cross-cultural differences in emotional expressivity and non-verbal behavior may further modulate transfer effectiveness and warrant explicit investigation in future work.

Sixth, while the present study extends prior work by incorporating cross-attention fusion, pairwise sequential integration was employed across three fusion stages. Joint transformer-based cross-modal attention across all modalities simultaneously may capture richer inter-modal dependencies that pairwise sequential integration necessarily misses, and is likely to benefit further from pretraining given the larger parameter space involved. This direction warrants future investigation, particularly as larger datasets become available.

Finally, sensitivity and specificity at the default threshold (≥0.5) for the best-performing cross-attention configuration were approximately 0.154 and 0.918, respectively, reflecting the inherent precision–recall trade-off under severe class imbalance. Clinical deployment would require threshold calibration aligned with the target use case (e.g., prioritizing sensitivity for screening). Formal calibration analysis is recommended for future work with larger sample sizes, where reliability curves can be estimated with sufficient stability.

## Figures and Tables

**Table 1:** Detailed comparison focusing on pretraining and fusion design. PT = pretraining; Interp. = interpretability.

Study	Modalities	Vision PT	Text PT	Audio PT	Fusion	Interp.	Transfer Analysis
Yang et al. (2024) [1]	V + A + T	No	No	No	Early	No	No
Li et al. (2025) [2]	V + A + T	Partial	Yes	No	Kronecker	No	No
Nykoniuk et al. (2025) [3]	A + T	–	Yes	No	Early/Late	No	No
Jia et al. (2025) [4]	A + EEG	No	No	No	Attention + GCN	No	No
**Proposed (Ours)**	V (SigLIP + Pose) + A + T	Yes (SigLIP)	Yes (E5/ WRIME)	Yes (JTES)	Concat/Sum/ Gated/Attention	Yes (Gate + Attn)	Yes (Controlled, 512 cond.)

**Table 2:** Comparison of the four fusion strategies evaluated in this study. Parameter overhead is reported relative to the shared hidden dimension d=256 per fusion stage.

Strategy	Operation	Extra Params	Weighting	Interpretability
Concatenation	$\mathrm{Linear}(\left[h_{a};h_{b}\right])\to h$	∼2$d^{2}$	Fixed	None
Summation	$h_{a}+h_{b}$	0	Equal	None
Gated	$g\cdot h_{a}+\left(1-g\right)\cdot h_{b}$	∼2$d^{2}+d$	Scalar gate	Gate weight *g*
Cross-Attention	$W_{O}\left[h_{a};\alpha V\right]$	∼4$d^{2}$	Soft attention	Attention map α

**Table 3:** AUC (±SD) for each combination of affective pretraining and auxiliary feature configuration (concat fusion).

Pretraining	None	E5	OpenSMILE	E5 + OpenSMILE
None	**0.747 ± 0.182**	0.720 ± 0.157	0.625 ± 0.109	0.582 ± 0.089
Mosi	**0.680 ± 0.171**	0.635 ± 0.138	0.594 ± 0.100	0.673 ± 0.192
Mosei	**0.826 ± 0.122**	0.814 ± 0.132	0.643 ± 0.093	0.673 ± 0.094

**Table 4:** Mean AUC per fusion strategy at each fusion position (*n* = 128 per cell). Kruskal–Wallis test conducted across four strategies within each position. Effect size reported as $\eta^{2}=H/\left(N-1\right)$.

Position	Strategy	AUC (Mean ± SD)	*H*/*p*/$\eta^{2}$
vis_fusion	Concat	**0.769 ± 0.035**	25.73/<0.001/0.203
	Sum	0.746 ± 0.036	
	Gated	0.746 ± 0.042	
	Attention	0.751 ± 0.042	
audio_fusion	Concat	0.764 ± 0.035	86.28/<0.001/0.679
	Sum	0.743 ± 0.031	
	Gated	0.733 ± 0.045	
	Attention	**0.774 ± 0.034**	
text_fusion	Concat	0.754 ± 0.034	0.89/0.828/0.007
	Sum	0.751 ± 0.037	
	Gated	0.752 ± 0.048	
	Attention	**0.756 ± 0.039**	

**Table 5:** Mean PR-AUC per fusion strategy at each fusion position (*n* = 128 per cell). Effect size reported as $\eta^{2}=H/\left(N-1\right)$.

Position	Strategy	PR-AUC (Mean ± SD)	*H*/*p*/$\eta^{2}$
vis_fusion	Concat	**0.583 ± 0.075**	19.58/<0.001/0.154
	Sum	0.554 ± 0.066	
	Gated	0.544 ± 0.069	
	Attention	0.551 ± 0.073	
audio_fusion	Concat	0.570 ± 0.060	101.99/<0.001/0.803
	Sum	0.536 ± 0.061	
	Gated	0.521 ± 0.068	
	Attention	**0.606 ± 0.069**	
text_fusion	Concat	0.551 ± 0.066	16.08/0.001/0.127
	Sum	0.549 ± 0.072	
	Gated	0.554 ± 0.084	
	Attention	**0.579 ± 0.062**	

**Table 6:** Effect of modality-specific pretraining on mean AUC (Mann–Whitney *U* test, n=256 per condition). $\Delta =\mu_{\mathrm{PT}}-\mu_{\mathrm{SC}}$, where PT denotes pretrained and SC denotes scratch initialization. ** p<0.01; *** p<0.001.

Modality	PT (Mean ± SD)	SC (Mean ± SD)	Δ	*p*	Effect
SigLIP (MOSI/MOSEI)	0.762±0.041	0.744±0.036	+0.018	<0.001 ***	Positive
Audio (JTES)	0.746±0.049	0.760±0.027	−0.014	0.004 **	Negative
Text (WRIME)	0.751±0.040	0.756±0.040	−0.005	<0.229	n.s.
All 3 PT vs. All SC	0.755±0.050	0.755±0.023	−0.000	—	

**Figure A1:**
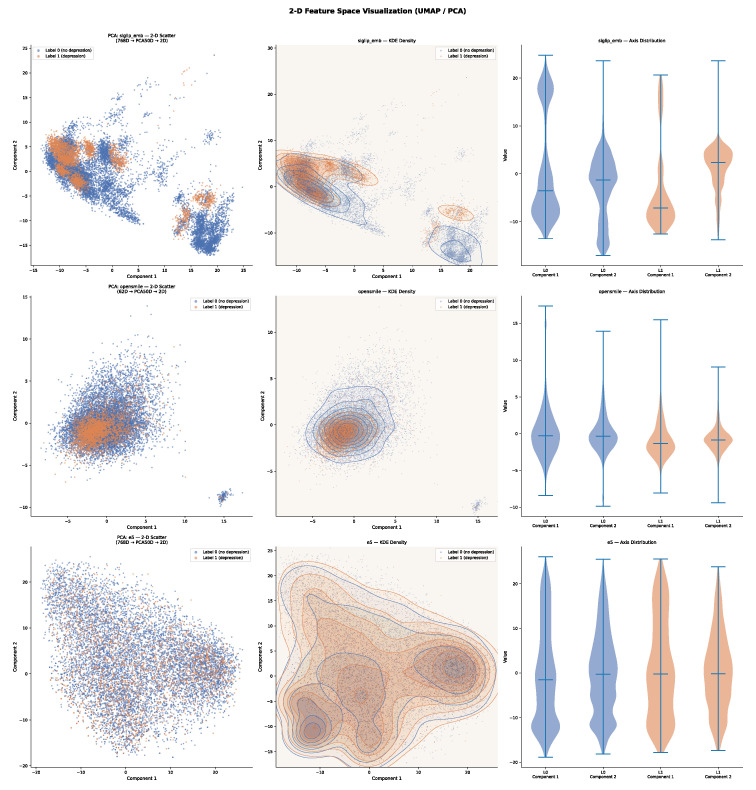
UMAP-based visualization of modality embeddings prior to fusion. Top row: SigLIP visual semantic embeddings. Middle row: OpenSMILE acoustic descriptors. Bottom row: E5 textual embeddings. Left: 2D UMAP projections colored by depression label. Center: Kernel density estimates (KDE) of projected distributions. Right: Label-wise distribution comparison along UMAP axes. Across modalities, clusters exhibit partial overlap between depressed and non-depressed groups, indicating that single-modality embeddings alone do not consistently provide clear separability. Notably, acoustic descriptors display broader dispersion due to high-dimensional handcrafted features. These observations support the necessity of dynamic fusion mechanisms that adaptively integrate complementary modality cues rather than relying solely on static concatenation.

## Data Availability

The original contributions presented in this study are included in the article. Further inquiries can be directed to the corresponding author.

## Supplementary Materials

The following supporting information can be downloaded at https://www.mdpi.com/article/10.3390/brainsci16040366/s1.

## Appendix A. Representation Geometry Analysis

To qualitatively examine modality-specific embedding geometry prior to fusion, we visualized per-utterance representations using UMAP dimensionality reduction. Figure A1 presents label-conditioned projections for SigLIP, OpenSMILE, and E5 embeddings.
