# Supplementary material for: Interaction-Driven Dynamic Fusion for Multimodal Depression Detection: A Controlled Analysis of Gating and Cross-Attention Under Class Imbalance
Source: Brain Sci. 2026 Mar 28;16(4):366. doi: 10.3390/brainsci16040366 (PMC13115347; doi:10.3390/brainsci16040366)
Supplement: Supplementary file 1 [file brainsci-16-00366-s001.zip › brainsci-4206857-supplementary.pdf]

Supplementary Materials

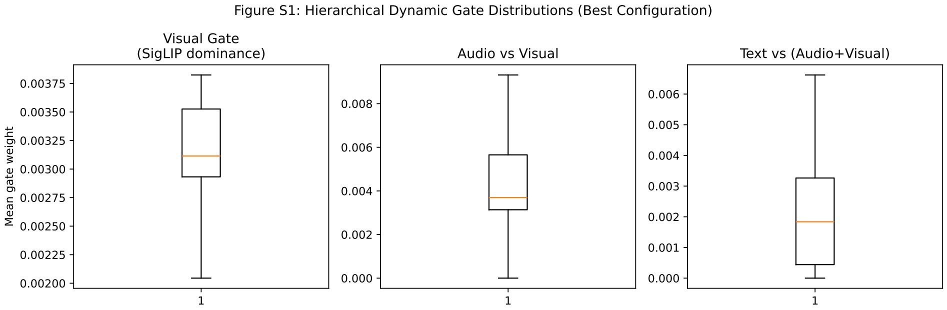

Figure S1: Hierarchical dynamic gate distributions under the best-performing cross-attention configuration. Left: intra-visual gate weighting between SigLIP semantic embeddings and head pose features. Center: audio-versus-visual cross-modal gate. Right: text-versus-(audio+visual) gate. Each point represents the session-level mean gate value averaged across utterances. The wide dispersion observed in cross-modal gates indicates subject-specific and context-dependent modality reweighting beyond static fusion assumptions.

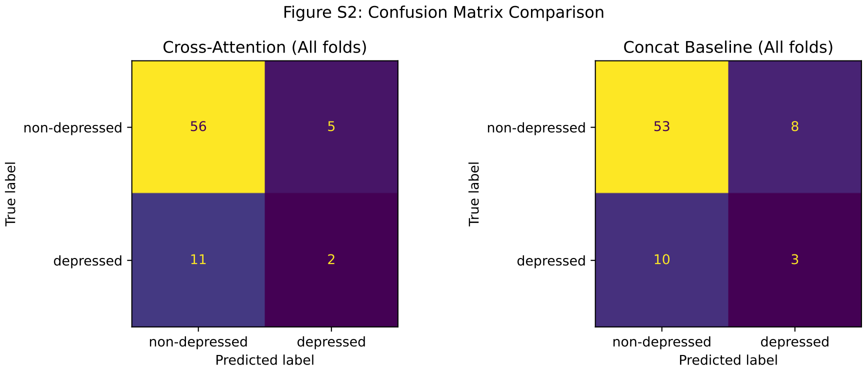

Figure S2: Confusion matrices aggregated across five folds for the best-performing cross-attention configuration (left) and the concatenation baseline (right). Cross-attention reduces false positives in the non-depressed group, while exhibiting a different error distribution for the depressed class. These differences reflect threshold-dependent trade-offs consistent with the PR-AUC analysis reported in Table 5.

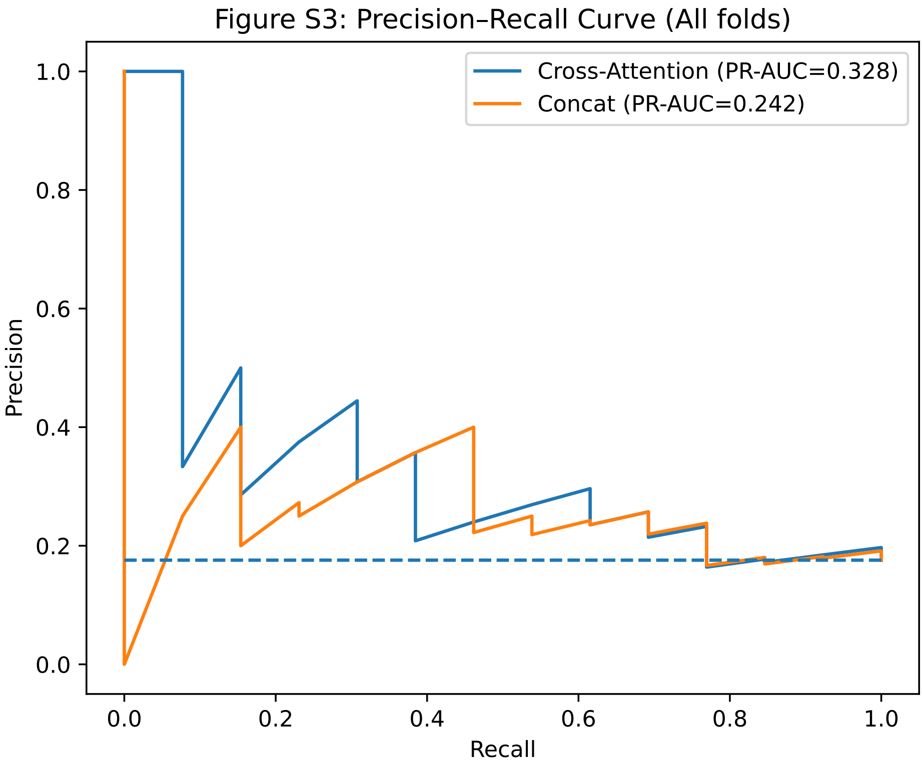

Figure S3: Precision–recall curves aggregated across five folds for the cross-attention model and the concatenation baseline. Cross-attention achieves a higher PR-AUC (0.328) than concatenation (0.242), indicating improved minority-class discrimination under class imbalance. The curve illustrates superior precision at moderate recall levels for the depressed class.
